# Complete genome sequences of 38 *Streptococcus sanguinis* human-associated isolates

**DOI:** 10.1128/mra.00546-26

**Published:** 2026-09-10

**Authors:** Martha A. Zepeda-Rivera, Sean L. Cotton, Seon-Sook An, Christopher D. Johnston, Todd Kitten

**Affiliations:** 1 Genomic Medicine, MD Anderson Cancer Center 4002, Houston, Texas, USA; 2 The Forsyth Institute, Microbiology 1816, Cambridge, Massachusetts, USA; 3 Department of Oral and Craniofacial Molecular Biology, School of Dentistry, Virginia Commonwealth University 6889, Richmond, Virginia, USA; University of Pittsburgh School of Medicine, Pittsburgh, Pennsylvania, USA

**Keywords:** *Streptococcus sanguinis*, endocarditis, PacBio, SMRT-Seq

## Abstract

We report the complete genome sequences of 38 *Streptococcus sanguinis* genomes, isolated from human oral and endocarditis-associated samples. These genomes are each comprised of a single chromosome that ranges from approximately 2.29 to 2.48 Mb in length, with an average GC content of 43.2%.

## ANNOUNCEMENT

**Fig 1:**
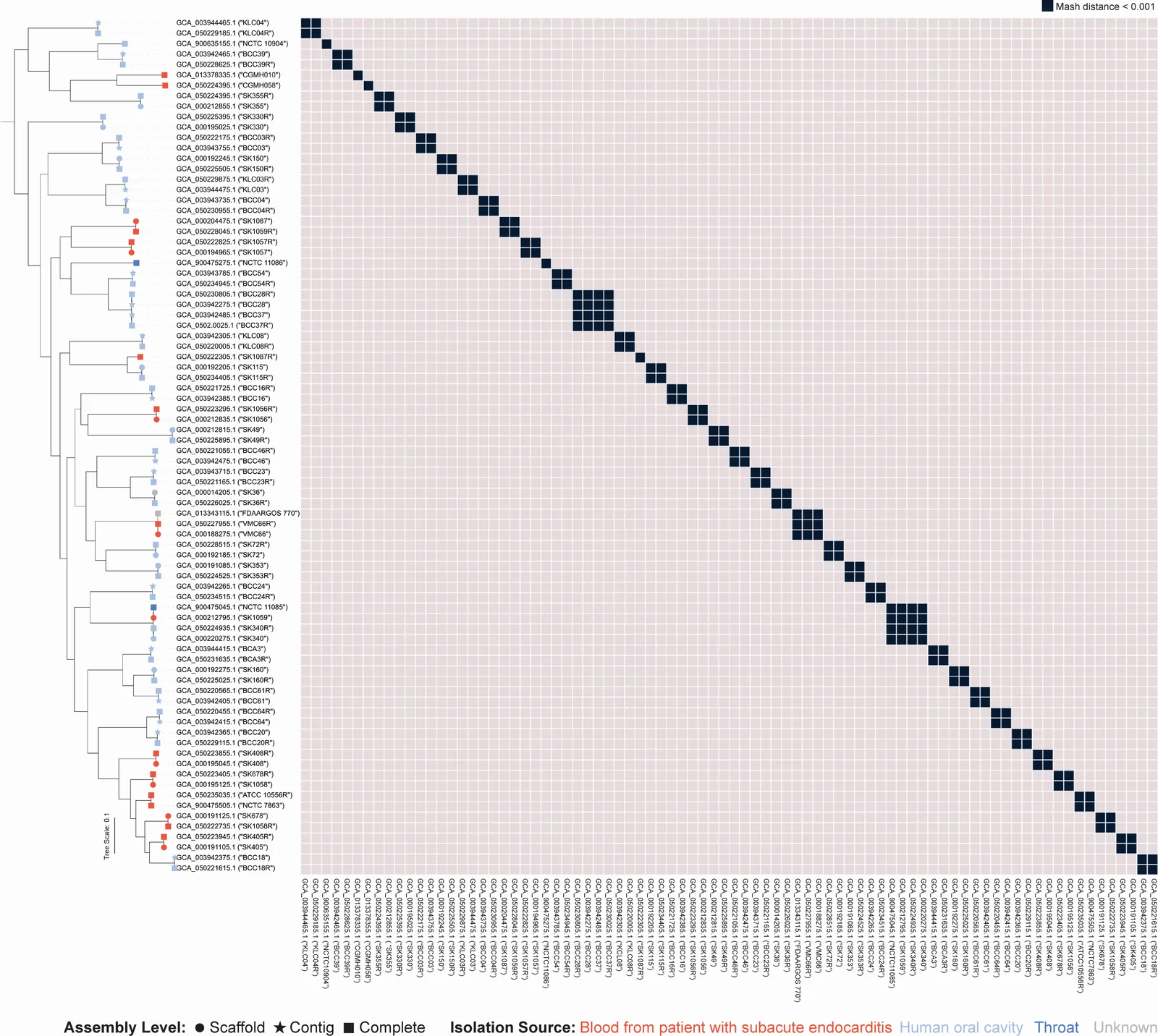
Mash distance between *Streptococcus sanguinis* genomes with a corresponding reference-free whole-genome phylogenetic tree. The figure displays (left) a kSNP4 (12) (kmer = 17, FCK = 0.348) whole-genome phylogenetic tree of available *Streptococcus sanguinis* genome assemblies (*N* = 82) with (right) corresponding Mash (13) distance values less than 0.001 shown in dark blue. Tree endpoint shape indicates assembly level, and color indicates isolation source. Mash distance heatmap was generated with pheatmap R package, RStudio version 2024.12.0+467. Tree was visualized using iTOL (14), and figure was made with Adobe Illustrator.

*Streptococcus sanguinis* is a gram-positive facultative anaerobe originally associated with infective endocarditis (1, 2). It resides predominantly in the human oral cavity where it is also associated with oral health (3–7). A barrier to the study of *S. sanguinis* as a pathogen or commensal has been the limited number of strains with completely sequenced genomes (Fig. 1). Here, we report the complete genome sequences of 38 *S. sanguinis* strains previously isolated from human oral or endocarditis-associated samples across Denmark, Greenland, Japan, and the United States (8–11; Table 1).

**TABLE 1:** *Streptococcus sanguinis* genome assemblies^*a*^

Strain	Accession	Length	No. contigs	Assembly level	Release date	GC %	Sequencing technology	Submitter	Country	Source	Mean coverage	BioProject	BioSample	Sequence Read Archive accession	No. subreads	N50
ATCC 10556R	GCA_050235035.1	2,297,357	1	Complete Genome	06-05-2025	43.5	PacBioSequel	Forsyth Institute	Unknown	Patient with subacute endocarditis	218×	PRJNA549513	SAMN25847823	SRR39526714	1,05,947	59,500
BCA3R	GCA_050231635.1	2,352,112	1	Complete Genome	06-05-2025	43.5	PacBioSequel	Forsyth Institute	USA	Human oral cavity	64×	PRJNA549513	SAMN19645995	SRR39526713	44,086	58,306
BCC03R	GCA_050222175.1	2,383,565	1	Complete Genome	06-05-2025	43.0	PacBioSequel	Forsyth Institute	USA	Human oral cavity	272×	PRJNA549513	SAMN18132949	SRR39526702	1,71,805	56,298
BCC04R	GCA_050230955.1	2,411,296	1	Complete Genome	06-05-2025	43.0	PacBioSequel	Forsyth Institute	USA	Human oral cavity	240×	PRJNA549513	SAMN19645996	SRR39526691	1,48,666	57,591
BCC16R	GCA_050221725.1	2,452,411	1	Complete Genome	06-05-2025	43.0	PacBioSequel	Forsyth Institute	USA	Human oral cavity	219×	PRJNA549513	SAMN18132950	SRR39526683	1,27,060	55,228
BCC18R	GCA_050221615.1	2,355,738	1	Complete Genome	06-05-2025	43.0	PacBioSequel	Forsyth Institute	USA	Human oral cavity	248×	PRJNA549513	SAMN18132951	SRR39526882	1,16,617	91,699
BCC20R	GCA_050229115.1	2,389,772	1	Complete Genome	06-05-2025	43.0	PacBioSequel	Forsyth Institute	USA	Human oral cavity	134×	PRJNA549513	SAMN19646001	SRR39526681	72,996	54,407
BCC23R	GCA_050221165.1	2,396,799	1	Complete Genome	06-05-2025	43.5	PacBioSequel	Forsyth Institute	USA	Human oral cavity	285×	PRJNA549513	SAMN18132952	SRR39526680	1,36,969	90,839
BCC24R	GCA_050234515.1	2,328,960	1	Complete Genome	06-05-2025	43.0	PacBio Sequel	Forsyth Institute	USA	Human oral cavity	221×	PRJNA549513	SAMN25847825	SRR39526679	1,12,923	60,331
BCC28R	GCA_050230805.1	2,395,963	1	Complete Genome	06-05-2025	43.0	PacBio Sequel	Forsyth Institute	USA	Human oral cavity	85×	PRJNA549513	SAMN19645997	SRR39526678	37,363	43,761
BCC37R	GCA_050230025.1	2,395,964	1	Complete Genome	06-05-2025	43.0	PacBio Sequel	Forsyth Institute	USA	Human oral cavity	122×	PRJNA549513	SAMN19645998	SRR39526712	73,757	54,364
BCC39R	GCA_050228625.1	2,431,669	1	Complete Genome	06-05-2025	43.0	PacBio Sequel	Forsyth Institute	USA	Human oral cavity	158×	PRJNA549513	SAMN19646002	SRR39526711	97,775	55,981
BCC46R	GCA_050221055.1	2,335,154	1	Complete Genome	06-05-2025	43.5	PacBio Sequel	Forsyth Institute	USA	Human oral cavity	185×	PRJNA549513	SAMN18132953	SRR39526710	85,777	91,425
BCC54R	GCA_050234945.1	2,482,700	1	Complete Genome	06-05-2025	43.0	PacBio Sequel	Forsyth Institute	USA	Human oral cavity	310×	PRJNA549513	SAMN25847824	SRR39526709	1,50,487	60,407
BCC61R	GCA_050220565.1	2,395,902	1	Complete Genome	06-05-2025	43.0	PacBio Sequel	Forsyth Institute	USA	Human oral cavity	214×	PRJNA549513	SAMN18132954	SRR39526708	1,02,484	90,718
BCC64R	GCA_050220455.1	2,397,254	1	Complete Genome	06-05-2025	43.0	PacBio Sequel	Forsyth Institute	USA	Human oral cavity	81×	PRJNA549513	SAMN18132955	SRR39526707	35,648	43,973
KLC03R	GCA_050229875.1	2,377,638	1	Complete Genome	06-05-2025	43.0	PacBio Sequel	Forsyth Institute	USA	Human oral cavity	206×	PRJNA549513	SAMN19645999	SRR39526705	1,32,592	67,937
KLC04R	GCA_050229185.1	2,344,317	1	Complete Genome	06-05-2025	43.0	PacBio Sequel	Forsyth Institute	USA	Human oral cavity	97×	PRJNA549513	SAMN19646000	SRR39526704	42,967	54,768
KLC08R	GCA_050220005.1	2,354,823	1	Complete Genome	06-05-2025	43.5	PacBio Sequel	Forsyth Institute	USA	Human oral cavity	202×	PRJNA549513	SAMN18132956	SRR39526703	1,57,301	62,460
SK1056R	GCA_050223295.1	2,384,203	1	Complete Genome	06-05-2025	43.0	PacBio Sequel	Forsyth Institute	Denmark	Blood from patient with infective endocarditis	256×	PRJNA549513	SAMN18132945	SRR39526700	1,44,591	90,587
SK1057R	GCA_050222825.1	2,356,393	1	Complete Genome	06-05-2025	43.5	PacBio Sequel	Forsyth Institute	Denmark	Blood from patient with infective endocarditis	326×	PRJNA549513	SAMN18132946	SRR39711331	1,90,973	90,219
SK1058R	GCA_050222735.1	2,307,179	1	Complete Genome	06-05-2025	43.5	PacBio Sequel	Forsyth Institute	Denmark	Blood from patient with infective endocarditis	288×	PRJNA549513	SAMN18132947	SRR39526699	1,52,821	90,310
SK1059R	GCA_050228045.1	2,327,597	1	Complete Genome	06-05-2025	43.0	PacBio Sequel	Forsyth Institute	Denmark	Blood from patient with infective endocarditis	272×	PRJNA549513	SAMN19646004	SRR39526698	1,32,571	90,442
SK1087R	GCA_050222305.1	2,344,952	1	Complete Genome	06-05-2025	43.5	PacBio Sequel	Forsyth Institute	Denmark	Blood from patient with infective endocarditis	233×	PRJNA549513	SAMN18132948	SRR39526697	1,19,532	90,425
SK115R	GCA_050234405.1	2,352,698	1	Complete Genome	06-05-2025	43.5	PacBio Sequel	Forsyth Institute	Greenland	Human oral cavity	374×	PRJNA549513	SAMN25847826	SRR39526696	1,79,061	60,222
SK150R	GCA_050225505.1	2,336,320	1	Complete Genome	06-05-2025	43.5	PacBio Sequel	Forsyth Institute	Denmark	Human oral cavity	454×	PRJNA549513	SAMN18132936	SRR39526695	2,16,049	70,374
SK160R	GCA_050225025.1	2,344,309	1	Complete Genome	06-05-2025	43.5	PacBio Sequel	Forsyth Institute	Denmark	Human oral cavity	333×	PRJNA549513	SAMN18132937	SRR39526694	1,62,044	70,068
SK330R	GCA_050225395.1	2,436,563	1	Complete Genome	06-05-2025	43.0	PacBio Sequel	Forsyth Institute	USA	Human oral cavity	509×	PRJNA549513	SAMN18132938	SRR39526693	2,99,676	70,972
SK340R	GCA_050224935.1	2,417,068	1	Complete Genome	06-05-2025	43.0	PacBio Sequel	Forsyth Institute	USA	Human oral cavity	552×	PRJNA549513	SAMN18132939	SRR39526701	3,28,406	70,895
SK353R	GCA_050224525.1	2,319,454	1	Complete Genome	06-05-2025	43.5	PacBio Sequel	Forsyth Institute	Japan	Human oral cavity	389×	PRJNA549513	SAMN18132940	SRR39526692	2,45,251	67,702
SK355R	GCA_050224395.1	2,386,238	1	Complete Genome	06-05-2025	43.5	PacBio Sequel	Forsyth Institute	Japan	Human oral cavity	668×	PRJNA549513	SAMN18132941	SRR39526690	3,75,613	69,567
SK36R	GCA_050226025.1	2,397,480	1	Complete Genome	06-05-2025	43.5	PacBio Sequel	Forsyth Institute	Denmark	Human oral cavity	93×	PRJNA549513	SAMN18132934	SRR39526689	37,913	32,787
SK405R	GCA_050223945.1	2,313,761	1	Complete Genome	06-05-2025	43.5	PacBio Sequel	Forsyth Institute	Denmark	Blood from patient with infective endocarditis	368×	PRJNA549513	SAMN18132942	SRR39526688	1,96,255	69,362
SK408R	GCA_050223855.1	2,418,848	1	Complete Genome	06-05-2025	43.0	PacBio Sequel	Forsyth Institute	Denmark	Blood from patient with infective endocarditis	407×	PRJNA549513	SAMN18132943	SRR39526687	2,03,697	70,760
SK49R	GCA_050225895.1	2,294,223	1	Complete Genome	06-05-2025	43.0	PacBio Sequel	Forsyth Institute	Denmark	Human oral cavity	124×	PRJNA549513	SAMN18132935	SRR39526686	48,079	33,551
SK678R	GCA_050223405.1	2,363,465	1	Complete Genome	06-05-2025	43.0	PacBio Sequel	Forsyth Institute	Denmark	Blood from patient with infective endocarditis	611×	PRJNA549513	SAMN18132944	SRR39526685	2,99,927	71,955
SK72R	GCA_050228515.1	2,395,017	1	Complete Genome	06-05-2025	43.5	PacBio Sequel	Forsyth Institute	Greenland	Human oral cavity	91×	PRJNA549513	SAMN19646003	SRR39526684	37,642	32,503
VMC66R	GCA_050227955.1	2,332,702	1	Complete Genome	06-05-2025	43.5	PacBio Sequel	Forsyth Institute	USA	Blood from patient with infective endocarditis	381×	PRJNA549513	SAMN19646005	SRR39526706	2,60,483	91,251

^*a*^ Names and associated metadata for the 38 *Streptococcus* genome assemblies released for and described in this work.

*S. sanguinis* strains were cultured in Brain Heart Infusion (BHI) broth for DNA isolation. Briefly, 5 μL of frozen aliquot was inoculated into 5 mL BHI and incubated at 37°C for 20 h. Cultures were diluted in pre-warmed BHI and grown for 1–3 h at 37°C. Cells were pelleted by centrifugation. High-molecular-weight DNA was extracted utilizing the MasterPure DNA Purification Kit (LGC Biosearch Technologies). DNA was sheared as previously described (15) and size-selected for fragments >5 kb using BluePippin (Sage Science). Sequencing libraries were prepared using the HiFi assembly kit (Pacific Biosciences). Single-molecule real-time sequencing (SMRT-Seq) (16) with base kinetics was performed on a PacBio Sequel I instrument (Pacific Biosciences). Sequencing reads were quality filtered and processed using Microbial Assembler in the Pacific Biosciences’ SMRTAnalysis pipeline (v9.0.0.92188) with default parameters, which determined contig circularization. All final assemblies were composed of a single circular chromosome that ranged from 2.29 to 2.48 Mb in length (average of 2.37 Mb) and 43.0%–43.5% in GC content (average of 43.2%) (Table 1). These final genome assemblies were classified as either *Streptococcus* or *Streptococcus sanguinis* via GTDB-tk (17, 18) (v2.3.2) using default parameters.

*S. sanguinis* strain or sequence misidentification has previously been reported (8). To corroborate strain identities and assess genome assembly duplicates, we performed phylogenetic (12) and Mash (13) analyses against publicly available *S. sanguinis* genomes. As all strains except ATCC 10556 already had contig-level sequences deposited in GenBank (8, 10, 19), we appended the letter “R” (for “Reference”) to each strain name to distinguish our complete assemblies, which we propose as the true reference for each strain. Twenty-nine of our genome assemblies displayed low Mash distance (<8.06^E−04^) to a single previously submitted genome sequence of the same name (minus the “R” designation). One assembly, SK1087R, had a low Mash distance only to itself. Eight assemblies showed low Mash distance (<3.13^E−04^) to strains with different identifiers forming seven groups; these included (i) ATCC 10556R and NCTC 7863; (ii) VMC66R, VMC66, and FDA-ARGOS_770; (iii) SK1059R and SK1087; (iv) SK678R and SK1058; (v) SK1058R and SK678; (vi) BCC28R, BCC37R, BCC28, and BCC37; and (vii) SK340R, SK340, SK1059, and NCTC 11085. While (i) reflects known synonymous identifiers, (ii) is consistent with FDA-ARGOS genome names (20), and (iii–vi) are consistent with previous reports (8), the relatedness in (vii) to NCTC 11085 (21) warrants further investigation.

The release of these 38 genome assemblies represents an approximately sixfold increase in publicly available, complete *S. sanguinis* genomes. Together with their corresponding physical isolates, these genomes will support future mechanistic studies of *S. sanguinis* as both a pathogen and commensal organism.

## Data Availability

The BioProject accession number for these genomes is PRJNA549513. Raw sequencing reads and assembled genomes were deposited in the National Center for Biotechnology Information, and corresponding raw read accession and BioSample numbers are listed in Table 1.
